# A Mathematical Modelling Approach for Systems Where the Servers Are Almost Always Busy

**DOI:** 10.1155/2012/290360

**Published:** 2012-04-11

**Authors:** Christina Pagel, David A. Richards, Martin Utley

**Affiliations:** ^1^Clinical Operational Research Unit, University College London, 4 Taviton Street, London WC1H 0BT, UK; ^2^Psychology, College of Life and Environmental Sciences, University of Exeter, Perry Road, Exeter EX4 4QG, UK

## Abstract

The design and implementation of new configurations of mental health services to meet local needs is a challenging problem. In the UK, services for common mental health disorders such as anxiety and depression are an example of a system running near or at capacity, in that it is extremely rare for the queue size for any given mode of treatment to fall to zero. In this paper we describe a mathematical model that can be applied in such circumstances. The model provides a simple way of estimating the mean and variance of the number of patients that would be treated within a given period of time given a particular configuration of services as defined by the number of appointments allocated to different modes of treatment and the referral patterns to and between different modes of treatment. The model has been used by service planners to explore the impact of different options on throughput, clinical outcomes, queue sizes, and waiting times. We also discuss the potential for using the model in conjunction with optimisation techniques to inform service design and its applicability to other contexts.

## 1. Introduction

Health treatment activities where arriving patients might have to wait for treatment and where duration of treatment follows a certain probability distribution have often been modelled using queueing theory. A classic example is the study of accident and emergency departments in acute hospitals [1, 2]. However in situations where “treatment” consists of a set of distinct treatment types, with the possibility of queues at each treatment stage, and the possibility of receiving a given type of treatment more than once, the use of queueing theory becomes very complex [3, 4].

**Figure 1:**
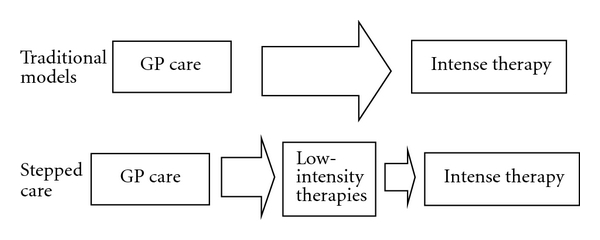
A diagram highlighting the difference between a traditional care model and the stepped care model. GP: General Practitioner.

The provision of mental health care for depression and anxiety in the primary care system is one such complex system. A configuration for mental health care delivery called “stepped care” is advocated for patients with common mental health problems [5, 6] to replace traditional systems (see Figure 1). Stepped care [7] is based on two principles: (a) “least burden,” so that an intervention received by a patient should be effective and appropriate whilst burdening the patient and the health care system as little as possible [8] and (b) “self-correction,” the provision of a system in place to detect lack of improvement, which in turn leads to alternative more intensive treatments being offered [9]. Thus, in a stepped care system, patients are typically first considered for low-intensity interventions such as guided self-help, group work, or a short course of individual therapy. High-intensity interventions typically involve many sessions with a highly trained professional, such as a cognitive behavioural therapist. There are many different types of both low-intensity and high-intensity interventions, and an individual patient can step both “up” to, or between, high intensity treatments and “down” to, or between, low-intensity treatments.

The introduction of stepped care within an existing mental health care framework is challenging. Planning the delivery of stepped care requires decisions concerning the treatments to be offered, the number and type of staff, the protocol for how patients transfer between treatments, and the balance of provision between low and high intensity treatments. The other key feature of mental health care systems other than their complexity is that they are often operating at capacity. This is more feasible than in acute hospital environments since patients in the queue effectively wait “at home” using little resource.

In this paper we describe a mathematical model we have developed to help planners design a stepped care mental health system [10, 11], by providing rapid estimates of throughput and changes in waiting times for different potential configurations. This model was implemented within a software tool that was distributed to pilot primary mental health care providers [11]. We note that the mathematics presented here, although discussed in the specific context of mental health care delivery, is intended to be generic and is suitable for many other systems that meet certain assumptions.

## 2. Methods: Mathematical Model

The approach used is complementary to traditional queueing theory and is most suited to systems where traffic intensities are greater than or equal to one or to a system where the starting states have large queues. It complements recent work on queueing systems where some of the servers are always busy [12]. Additionally, this analysis is not dependent on the distributions of arrivals or duration of treatment. We note that where the traffic intensity is greater than one, there is no mathematical steady state to the system (since queues will increase indefinitely). However, the features of the system can still be explored within a specified time frame to understand better the distribution of demand between servers and the potential impact of increased or redistributed capacity.

### 2.1. A Single Treatment Slot

#### 2.1.1. Assumptions

The unit of capacity we consider in this analysis is a time slot in a diary (e.g., a therapy session) and we assume that patients are treated in discrete sessions. A given time slot in a diary is assumed to be devoted to one, and only one, distinct treatment type. We further assume that a patient takes at least one session to be treated, and that at the start of the modeled period there is no patient currently undergoing treatment (i.e., at *t* = 0 the time slot is either empty or a patient has just started their treatment). We assume that durations of treatment of different patients are independent of one another.

#### 2.1.2. Notation

For *x* ≥ 1, let *p*_*x*_ denote the probability that a patient's treatment time is exactly *x* time units. Define *p*_0_ = 0.

For *x* ≥ 1, let *s*_*x*_ denote the probability that a patient's treatment time is strictly longer than *x* time units. Define *s*_0_ = 1.

For *i* ≥ 1, *t* ≥ 1 let *r*_*i*,*t*_ be the probability that exactly *i* patients have completed their treatment and that no other patient has started their treatment by time *t*. Define *r*_*i*,0_ = 0 for *i* ≥ 1.

For *i* ≥ 1, *T* ≥ 1, let *f*_*i*,*T*_ be the probability that at time *T* exactly *i* people have completed their treatment. Note that another patient may have started. Define *f*_*i*,0_ = 0 for *i* ≥ 1.

#### 2.1.3. The Distribution for the Number of People Who Have Completed Treatment by Time *T*

We begin by considering *r*_1,*t*_, the probability that by time *t* ≥ 1 exactly one person has arrived and left and no one else has yet started

(1) $$r_{1,t}=p_{t}.$$

We can then define *r*_*i*,*t*_ iteratively:

(2) $$r_{i,t}=\overset{t}{\underset{k=1}{\sum }}r_{i-1,k}r_{1,t-k}=\overset{t}{\underset{k=1}{\sum }}r_{i-1,k}p_{t-k}.$$

Thus we have derived an expression for the probability that at some time *t*, *i* people have been treated and no one else has yet started. To relax this latter condition, we now consider *f*_*i*,*T*_, the probability that at some time *T*, exactly *i* people have completed their treatment. We use *r*_*i*,*k*_ to calculate this and for a given time *T*:

(3) $$f_{i,T}=\overset{T}{\underset{k=1}{\sum }}r_{i,k}s_{T-k}.$$

### 2.2. A Network of Treatment Slots

We now extend the concept of a single treatment slot to a network of treatment slots that can be thought of as representing a given system.

#### 2.2.1. Notation

Consider a treatment slot of type *i*, for *i* = 1 ⋯ *L* and absorbing exit states of type *i*, for *i* = *L* + 1 ⋯ *M*. There are a constant number, *N*_*i*_, of each type of treatment slot or exit state for *i* = 1 ⋯ *M* where *N*_*i*_ = 1 for *i* = *L* + 1 ⋯ *M*.

Let *α*_*ij*_ be the probability that a person leaving a slot of type *i*, for *i* = 1 ⋯ *M* goes to a treatment slot or exit state of type *j* for *j* = 1 ⋯ *M*. Define *α*_*ij*_ = 0, for all *i* = *L* + 1 ⋯ *M* and *j* = 1 ⋯ *M* (i.e., no one leaves an exit state).

Define the random variable *X*_*i*_ as the number of people who have left a single treatment slot of type *i*, in a given time period for *i* = 1 ⋯ *L*. The expectation and variance of *X*_*i*_ are denoted *E*(*X*_*i*_) and Var⁡(*X*_*i*_), and we assume that these quantities are well defined.

Let *λ*_*i*_ be the Poisson arrival rate from outside the system to a slot of type *i*. Define *λ*_*i*_ = 0, for all *i* = *L* + 1 ⋯ *M* (i.e., no arrivals to exit states from outside the system).

Define the random variable *W*_*ij*_ as the number of people who have arrived at the queue for any slot of type *j* from a single treatment slot *i*, in a given time period for *j* = 1 ⋯ *M* and *i* = 1 ⋯ *L*.

Define the random variable *Y*_*ij*_ as the number of people who have arrived at the queue for any slot of type *j* from all *N*_*i*_ treatment slots *i*, in a given time period for *j* = 1 ⋯ *M* and *i* = 1 ⋯ *L*. 

Define the random variable *Y*_*j*_ as the number of people who have arrived at the queue for any slot of type *j* in a given time period for *j* = 1 ⋯ *M*.

Define *B*(*p*) as the Bernouilli distribution with parameter *p*, where 0 ≤ *p* ≤ 1.

Define *G*_*Y*_(*s*) as the generating function associated with any given probability distribution *Y*.

#### 2.2.2. General Results from Probability Theory

For a constant number *N*_*i*_ of independent random variables *X*_*i*_,

(4) $$\begin{array}{c} E\left(\overset{N_{i}}{\underset{k=1}{\sum }}X_{i}\right)=\overset{N_{i}}{\underset{k=1}{\sum }}E\left(X_{i}\right)=N_{i}E\left(X_{i}\right),\\ \mathrm{Var}\left(\overset{N_{i}}{\underset{k=1}{\sum }}X_{i}\right)=\overset{N_{i}}{\underset{k=1}{\sum }}\mathrm{Var}\left(X_{i}\right)=N_{i}\mathrm{Var}\left(X_{i}\right). \end{array}$$

If a positive integer-valued distribution *Z* has generating function *G*_*Z*_(*s*) and a probability distribution *Y* has generating function *G*_*Y*_(*s*) then the distribution *W* = ∑_*k*=1_^*Z*^ *Y* has generating function:

(5) $$G_{W}\left(s\right)=G_{Z}\left(G_{Y}\left(s\right)\right).$$

Additionally the expectation and variance of *Y* are given by

(6) $$\begin{array}{c} E\left(Y\right)=G_{Y}^{\prime }\left(1\right),\\ \mathrm{Var}\left(Y\right)=G_{Y}^{\prime \prime }\left(1\right)+E\left(Y\right)-E^{2}\left(Y\right). \end{array}$$

Proof of these results can be found in Grimmett and Stirzaker [13].

### 2.3. Flows through a Treatment in a General Network

**Figure 2:**
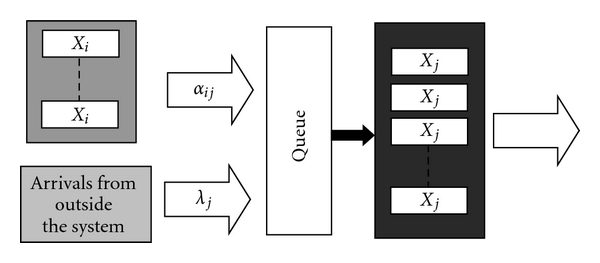
Flows in and out of treatment slots of type *j*.

Consider a treatment in a general network as shown in Figure 2. Here we consider flows in and out of a constant number, *N*_*j*_, of units of capacity of type *j*. In this “always full” system, people arriving at a treatment slot of type *j* will first enter a queue of unlimited size. The number of people in a queue waiting to enter any treatment slot of type *j* is denoted *Q*_*j*_. In what follows, we assume that there is no balking, but balking could be added to the system by specifying a maximum queue size.

As shown in Figure 2, flows into the queue for any slot of type *j* can come from either other units of capacity of type *i* ≠ *j* or from outside the system for *j* = 1 ⋯ *L*. In the special case of an exit state, there are no external arrivals and only one state of each type.

#### 2.3.1. Inputs from a Treatment Slot of Type *i*

For each person leaving a particular treatment slot of type *i*, for *i* = 1 ⋯ *L*, we can consider their destination as a Bernouilli trial, where they will enter the queue for a slot of type *j* with probability *α*_*ij*_. Over a given time period, we thus have a random number *X*_*i*_ of Bernouilli trials. Then

(7) $$W_{ij}=\overset{X_{i}}{\underset{k=1}{\sum }}B_{k}\left(\alpha_{ij}\right).$$

From (5) and (6) we obtain the following (a more detailed derivation is given in the appendix):

(8) $$\begin{array}{c} E\left(W_{ij}\right)=\alpha_{ij}E\left(X_{i}\right),\\ \mathrm{Var}\left(W_{ij}\right)={\alpha_{ij}}^{2}\mathrm{Var}\left(X_{i}\right)+\alpha_{ij}\left(1-\alpha_{ij}\right)E\left(X_{i}\right). \end{array}$$

Equation (8) give the expectation and variance for the total number of people who have arrived at the queue for a treatment slot of type *j* from a particular treatment slot of type *i* over the given time period. However, we have a block of *N*_*i*_ units of capacity of type *i* so we use (4) to derive the total number of people, *Y*_*ij*_, who arrive at the queue for any treatment slot of type *j* from the block of units of capacity of type *i* over the given time period:

(9) $$\begin{array}{c} E\left(Y_{ij}\right)=N_{i}\alpha_{ij}E\left(X_{i}\right),\\ \mathrm{Var}\left(Y_{ij}\right)=N_{i}\left({\alpha_{ij}}^{2}\mathrm{Var}\left(X_{i}\right)+\alpha_{ij}\left(1-\alpha_{ij}\right)E\left(X_{i}\right)\right). \end{array}$$

Thus

(10) $$\begin{array}{c} E\left(Y_{j}\right)=\lambda_{j}+\sum_{i\neq j}\alpha_{ij}N_{i}E\left(X_{i}\right),\\ \mathrm{Var}\left(Y_{j}\right)=\lambda_{j}+\sum_{i\neq j}\left({\alpha_{ij}}^{2}N_{i}\mathrm{Var}\left(X_{i}\right)+\alpha_{ij}\left(1-\alpha_{ij}\right)N_{i}E\left(X_{i}\right)\right), \end{array}$$

where the sum is over all different types of treatment slot *i*, *i* = 1 ⋯ *L*. Note that *α*_*ij*_ and *λ*_*j*_ can equal zero.

In circumstances where the network is always full, the output from units of capacity of type *j* for *j* = 1 ⋯ *L* is not dependent on the input into the queue. Thus the expected output from units of capacity of type *j* for *j* = 1 ⋯ *L* is

(11) $$\begin{array}{c} E\left(\text{out}\right)=N_{j}E\left(X_{j}\right),\\ \mathrm{Var}\left(\text{out}\right)=N_{j}\mathrm{Var}\left(X_{j}\right). \end{array}$$

We are now in a position to consider the expectation and variance of the change in the queue size *Q*_*j*_:

(12) $$\begin{array}{cc} E\left(\Delta Q_{j}\right) & =\sum_{i\neq j}\alpha_{ij}N_{i}E\left(X_{i}\right)+\lambda_{j}-N_{j}E\left(X_{j}\right),\\ \mathrm{Var}\left(\Delta Q_{j}\right) & =\sum_{i\neq j}\left({\alpha_{ij}}^{2}N_{i}\mathrm{Var}\left(X_{i}\right)+\alpha_{ij}\left(1-\alpha_{ij}\right)N_{i}E\left(X_{i}\right)\right)\\ & \;+\lambda_{j}+N_{j}\mathrm{Var}\left(X_{j}\right). \end{array}$$

Note that the variance of the change in queue size can be very large if there are a large number of potential inputs for that particular type.

For a patient waiting to receive treatment for a mental health problem, waiting time in a queue is more likely to be of concern than the actual number of people waiting. Let 1/*μ*_*j*_ represent the mean number of sessions required to treat a patient in a unit of capacity of type *j* for *j* = 1 ⋯ *L*. We can estimate the change in waiting time, Δ*P*_*j*_ for an individual arriving in the queue *Q*_*j*_ as

(13) $$\Delta P_{j}=\frac{E\left(\Delta Q_{j}\right)}{\mu_{j}N_{j}},\;j=1\cdots L.$$

### 2.4. Potential for Optimisation

The linear nature of the equations above in terms of *N*_*j*_ suggests the possibility that linear programming techniques might be used to optimise the configuration of the stepped care system according to some relevant criteria. An illustrative optimisation is given below where there are a set of treatment types *j* and a desired outcome *k* (for instance successful discharge) and the intention is to find the allocation of sessions to types of treatments that maximises the number of people achieving the desired outcome. We note that different objective functions can be defined and that optimisation functionality was not included in the software tool [11] produced as part of this project.

#### 2.4.1. Objective Function

Maximise:

(14) $$Z=\overset{}{\underset{j\neq k}{\sum }}\alpha_{jk}N_{j}E\left(X_{j}\right),\;j=1\cdots L,\;L<k\leq M.$$

#### 2.4.2. Constraints

1. *N*_*j*_ are positive integers, *j* = 1 ⋯ *L*
2. Total number of therapy sessions per week: ∑_*j*=1_^*L*^ *N*_*j*_ ≤ *S* for some integer *S*.
3. Total number of sessions for a treatment of type *j* is dependent on number of therapists qualified to deliver that type of treatment and thus there are capacity constraints for each treatment type: *N*_*j*_ ≤ *S*_*j*_, where *S*_*j*_ are positive integers, *j* = 1 ⋯ *L*.
4. Specify a maximum increase in waiting time of *W* weeks at each step:
  (15) $$E\left(\Delta Q_{j}\right)\leq WN_{j}\mu_{j},\;j=1\cdots L.$$

## 3. Results: Illustrative Example

This mathematical model has been implemented as part of a project examining the implementation of stepped care systems [11]. Here we give an illustrative example of its use on a hypothetical mental health care system and the subsequent potential for optimisation.

In this system there are three types of appointments available: an initial screening appointment, a low-intensity therapy appointment, and a high-intensity therapy appointment. People are either referred by their GP (General Practitioner) into the system in which case they begin with an assessment appointment or they can self-refer directly to low-intensity therapy treatment.

The proportion of patients moving between different types of appointment is shown in Table 1.

The health system managers have 100 weekly appointments available to cover all types of appointment. For the purposes of this example, we assume that the number of weekly booked sessions a patient uses for each type of treatment is exponential with means of 1, 3, and 6 for assessments, low-intensity and high-intensity sessions respectively. The arrivals and capacity allocation for the current system are given in Table 2.

Although the waiting times for screening and high-intensity treatments are acceptable, the increase in waiting time for a low-intensity appointment over 6 months is unacceptably long (12 weeks) (see columns 2 and 3 of Table 3). The manager wishes to optimise the allocation of appointment slots to appointment types to maximise the number of patients who successfully complete treatment in a 26-week period, according to the following constraints.

1. There can be a maximum of 100 total allocated appointments.
2. The maximum increase in average waiting time for an assessment is 4 weeks.
3. The maximum increase in average waiting time for either low or high intensity treatment is 8 weeks.
4. There must be at least 20 assessment sessions, 30 low-intensity, and 20 high-intensity sessions every week.

We ran this optimisation problem using Microsoft Excel Solver (version 2003). We note that Microsoft Solver is a standard add-in to Microsoft Excel and there exist several resources on its use within Excel (e.g., [14]). The new allocation and the output parameters calculated using the model are given in columns 4 and 5 of Table 3.

The suggested appointment schedule has resulted in a more even distribution of the expected waits for each type of treatment and increased the expected total number of people completing treatment over the 6-month time frame. 

## 4. Limitations

A clear limitation is the assumption that the system is always busy. However, application of the model to any given system would still provide the maximum possible throughput of the system over a given period of time. In the context of a mental health system, other limitations apply. Firstly, all patients are considered to be homogeneous and no allowance is made for patients with different characteristics (for instance presenting problem) having different duration of stay distributions or different pathways through the system. Secondly, in this analysis, time is considered to be defined by the number of treatment slots and thus application to a system where sessions are not regularly spaced in time is more complicated. Finally, “holding” or “blocking back” behaviour is not accounted for in the model, in that both the duration of treatment and the destination of patients from each treatment are assumed independent of the state of the system. 

## 5. Discussion

This mathematical model was developed in response to a specific problem within the configuration of mental health care services [10]. As part of that project, the model (without the optimisation aspect) has been implemented within a software tool developed to help planners explore the consequences of different configurations for a given mental health service. Details of the software tool and its use in designing stepped care systems can be found in the project final report [11]. 

We have described a simple way of analysing throughput and flows for a networked system in the situation where a system is always busy or where this is a reasonable approximation. We note that this is not a steady-state model and instead considers changes in mean output, queue sizes and waiting times over a relatively short (6 months) time period. We have also shown how optimisation techniques might be applied to the subsequent design of a network in the context of a mental health system. 

This approach could be useful in other health systems where “servers” (whether beds, clinicians or other resources are always busy) but a key assumption that needs to be met is that there will always be a queue. In practice this assumption is less likely to be valid for systems where queues involve people waiting in a physical allocated space (for instance, in an emergency department) than where people can “virtually” wait at home. Nonetheless, considering such busy systems over a short amount of time using these sorts of models can provide insight into the allocation of resources and management of arrivals to complement standard steady state queuing theory. 

## Figures and Tables

**Table 1:** Flows of patients between different types of appointment and two endpoints of the stepped care system.

From*∖*To	Assessment	Low intensity	High intensity	Completed treatment	Dropped out of treatment
Assessment	0%	40%	20%	10%	30%
Low intensity	0%	0%	20%	50%	30%
High intensity	0%	0%	0%	80%	20%

**Table 2:** Arrivals to the system and allocation of resources within the system.

Appointment type	Average number of new, external arrivals every week	Weekly appointment slots allocated
Assessment	20	30
Low intensity	10	40
High intensity	0	30

**Table 3:** Example use of optimisation to allocate available treatment slots to treatment types.

Appointment type/End point	Current weekly appointment slots allocated	Average increase in waiting time (weeks)	Suggested weekly appointment slots allocated	Average increase in waiting time (weeks)
Assessment	30	1.3	26	4
Low intensity	40	11.7	45	6
High intensity	30	7	29	7
*Dropped out*	*257*	—	*248 *	—
**Completed **	**292**	—	**301 **	—

## Appendix

Remember that the random variable *W*_*ij*_ is defined as the number of people who have arrived at the queue for any slot of type *j* from a single treatment slot *i*, in a given time period for *j* = 1 ⋯ *M* and *i* = 1 ⋯ *L*.

For each person leaving a particular treatment slot of type *i*, for *i* = 1 ⋯ *L*, we can consider their destination as a Bernouilli trial, where they will enter the queue for a slot of type *j* with probability *α*_*ij*_. Over a given time period, we thus have a random number *X*_*i*_ of Bernouilli trials. Then

(A.1) $$W_{ij}=\overset{X_{i}}{\underset{k=1}{\sum }}B_{k}\left(\alpha_{ij}\right).$$

From (5) we know that

(A.2) $$G_{X_{ij}}\left(s\right)=G_{X_{i}}\left(G_{B}\left(s\right)\right).$$

and thus

(A.3) $$\begin{array}{c} G_{X_{ij}}^{\prime }\left(s\right)=G_{X_{i}}^{\prime }\left(G_{B}\left(s\right)\right)G_{B}^{\prime }\left(s\right),\\ G_{X_{ij}}^{\prime \prime }\left(s\right)=G_{X_{ij}}^{\prime \prime }\left(G_{B}\left(s\right)\right){\left(G_{B}^{\prime }\left(s\right)\right)}^{2}+G_{X_{i}}^{\prime }\left(G_{B}\left(s\right)\right)G_{B}^{\prime \prime }\left(s\right). \end{array}$$

It is a standard result that *G*_*B*_(*s*) = (1 − *α*_*ij*_) + *α*_*ij*_ *s*, and so using this and (6) we obtain the following:

(A.4) $$\begin{array}{c} E\left(W_{ij}\right)=\alpha_{ij}E\left(X_{i}\right),\\ \mathrm{Var}\left(W_{ij}\right)={\alpha_{ij}}^{2}\mathrm{Var}\left(X_{i}\right)+\alpha_{ij}\left(1-\alpha_{ij}\right)E\left(X_{i}\right). \end{array}$$
